# Adapting Instead of Reacting: A Qualitative Study Exploring Parenting Strategies for Childhood Emotional Disturbance

**DOI:** 10.3390/children13020300

**Published:** 2026-02-21

**Authors:** Michelle L. Nighswander

**Affiliations:** St. David’s School of Nursing, Texas State University, Round Rock, TX 78665, USA; mln78@txstate.edu; Tel.: +1-512-408-2556

**Keywords:** parenting, childhood emotional disturbance, emotional or behavioral disorder, adaptation

## Abstract

**Highlights:**

**What are the main findings?**

- A consequence- or reaction-based parenting approach was universally ineffective for children with emotional disturbance.
- Prioritizing the adaptation of the child’s environment, defusing emotions, and pre-planned intentional responses were more successful in managing the child’s reactions and behaviors.

**What are the implications of the main findings?**

- Parents need healthcare providers to assist them in thinking of creative adaptations of the child’s environment rather than trying to fit the child within their environment.
- When mothers started seeing the child as struggling with a problem, rather than being simply defiant, mothers approached emotionally laden situations with compassion over frustration.

**Abstract:**

Background: Children with emotional disturbance (ED) frequently display highly unpredictable behaviors compared to other children. The magnitude and unpredictability of childhood ED make finding effective management strategies difficult for parents. Prior research has examined parents’ stress and the children’s behaviors in schools, but we know very little about how parents manage at home. Methods: This qualitative study used Naturalistic Inquiry to explore how parents respond to the challenges which arise at home due to childhood ED. Eight mothers raising 10 children with ED were recruited nationally. Data were gathered through semi-structured, individual interviews. Results: Consequences-based parenting strategies were unsuccessful, but mothers achieved greater success with pre-planned, intentional responses and adapting the child’s environment. Mothers learned their child’s world view was very different than their own. This realization caused mothers’ perspective toward their child to change. Mothers saw their child as struggling with a problem, instead of simply being defiant. The perception shift allowed mothers to approach situations with greater compassion and inner peace. Conclusions: The findings provide suggestions for pediatric healthcare providers who work with such parents seeking assistance and advice.

## 1. Introduction

Children with emotional disturbance frequently display unpredictable and disproportionate emotional and behavioral reactions, which present great challenges to their parents and families. Children with emotional disturbance (ED) simply react differently to circumstances than other children do [1]. It is difficult to adequately convey the sheer magnitude of the child’s unpredictability or the problems the child’s behaviors can create. Parents describe their child as “a volcano going off”, [2] (p. 4) or an explosion [3]. Some children become violent, causing property damage or threatening family members [4]. The children’s unpredictability makes finding effective management strategies difficult for parents.

In the literature, serious childhood emotional or behavioral problems may be identified by different names, such as “emotional disturbance”, “serious emotional disturbance”, or “emotional or behavioral disorder” [5]. Healthcare and social sciences researchers tend to utilize the terms “serious emotional disturbance” or “emotional or behavioral disorder”. The Diagnostic and Statistical Manual of Mental Disorders (DSM–5) considers “serious emotional disturbance” to be an adjunct condition that may accompany another diagnosed psychiatric illness [6]. This article uses the term “emotional disturbance”, which the United States Department of Education (DoEd) defines as functional, observable barriers to learning. According to the DoEd, a child may have an emotional disturbance if they demonstrate one of the following: an unexplained inability to learn, an inability to build non-familial relationships, inappropriate behaviors or feelings in normal circumstances, a persistent unhappiness, or develop health symptoms or fears from school issues [7]. The child’s behaviors must be present for at least three months, and must be so serious that they impair the child’s or other classmates’ learning. Unlike the DSM- 5 definition, “emotional disturbance” does not require that the child has another medically diagnosed psychiatric condition [7]. Regardless of the diagnostic term, these children display severe, unpredictable reactions that are dramatically out of proportion to the current circumstances, creating serious challenges at home and at school [8].

Children with uncontrolled emotional disturbance (ED) can create chaos both in and outside of the classroom [9]. The educational risks are high: twice as many children with ED are suspended (29%) as the general population, and children with ED are more likely to drop out of school altogether [10]. Educators spend significant energy and resources to manage the child’s behaviors in the school setting [9]. The problems caused by ED do not end with the school day. In addition to poor educational outcomes, children with ED experience high rates of failed relationships, unemployment, substance abuse, and incarceration [10,11]. By age 25, 60% of children with ED have been arrested at least once [12]. Parents of children with ED are obviously frightened that their child might become one of these statistics, and multiple studies have confirmed that parents of children with ED are highly stressed compared with other parents [13,14,15].

Parents of children with ED report high levels of stress and unmet needs, but there is little data describing how families manage daily challenges at home. Parents desire greater communication, education, and support to assist their child and their entire family [2,3,16,17]. Parents of children with ED report begging for help, stating they need more than “coffee and Valium!” [2] (p. 6), that “nothing works”, [4] (p. 196), and that “no one is telling us what we should do!” [3] (p. 169). As far back as 1999, the United States’ Surgeon General urged professionals to move beyond a traditional focus on the patient/child, and “embrace the family context” [18]. Paley and Hajal [19] advocated for researchers to consider emotional regulation to be a family-level process, rather than focusing on the child or the parent–child relationship exclusively. Family-level resilience is dependent on parental coping and availability of support, especially in families of children with emotional or behavioral disorders [20]. Several international, family-based training programs have been developed for parents of children with ED, including in Spain [21], Ireland [22], Sweden [23], and Finland [24]. These programs were all received positively and were noted to improve both child behaviors and general family function.

Unfortunately, parental support, training, or guidelines are only intermittently available in the United States. One Massachusetts program focused on improving caregivers’ mental health as well as child wellbeing within a family-centered service model [25]. An intensive home treatment program in Connecticut demonstrated significant improvement in functioning and a decrease in problem severity in youth with ED [26]. A school-based education and support program was piloted for parents of children with ED in 2005. The program was rated positively by participants, but was not continued [27]. The programs all indicate the potential of community-based success.

In addition to training programs, a few researchers examined methods for parental support. Personal self-care for parents of children with ED has been suggested in “mindful-based acceptance” [28] and practicing “self-compassion” [15]. Parent-to-parent support groups for parents of children with ED have been piloted through schools’ special education departments [13,29]. One case study advocated intentional “time in/time out” periods, which paired de-escalation “time outs” with periods of focused parental attachment [30]. A unique qualitative study used sensory modulation to create positive family dynamics between the child and the family members [31].

Despite the existence of evidence-based practices to support families of children with ED, the utilization rates in communities remain low [32]. Parents concerned about their child’s behaviors usually turn to pediatric experts first for guidance, but come away feeling frustrated and unheard [16,33]. A meta-analysis concluded that the primary care level is the best option to deliver parenting interventions with positive, equitable, and sustained impact at the population level [34]. Unfortunately, pediatricians struggle to advise parents of children with behavioral problems, with only 13% of pediatricians reporting confidence in their ability to manage behavioral problems in children [35]. Parents ask pediatric providers for tangible ideas to manage their child with ED and meet their other children’s needs; however, pediatric providers may struggle to provide effective advice for parents of children with ED.

Parents of children with ED desperately seek information and ideas, but there is a lack of knowledge regarding parenting strategies aimed toward managing a child with ED in the home setting. This study sought to learn from parents of children with ED what strategies parents tried, which strategies were successful, which strategies failed, and if parents’ strategies changed as the child grew older. By learning more about the strategies parents have used, successful or not, we may discover new ideas to share with other parents or research further. To reduce this knowledge gap, this study used Lincoln and Guba’s Naturalistic Inquiry methodology [36] to explore the methods and strategies currently used by mothers of children with ED to manage their child’s condition, and whether those strategies evolved over time.

## 2. Methods

### 2.1. Study Aim and Design

The aim of this study was to explore parents’ child-rearing experiences as related to the emotional or behavioral issues caused by their child’s emotional disturbance (ED). The study was guided by the following questions:

(1) How do parents respond to situations and challenges that arise due to their child’s ED?
(2) How do situations and parents’ responses change as the child matures?

The responses to these questions will give pediatric professionals insight about how parents attempt manage their child’s condition at home, which strategies were effective, and how parents’ perspectives and approaches change as a child with ED matures.

The lack of data surrounding parents’ experiences at home suggested the need for an exploratory qualitative approach. Lincoln and Guba’s Naturalistic Inquiry (NI) [36], as described by Erlandson et al. [37], was selected because NI focuses on gathering data surrounding the context and personal perspectives of persons experiencing a phenomenon. This allows researchers to construct a composite of the group’s reality and “see what is happening” within the problem of focus [37] (p. 9). Outside of being highly stressed, we have little information that illuminates parents’ experiences at home raising a child with ED. “Seeing what is happening” at home from mothers’ perspectives will assist professionals in offering improved advice and targeted ideas for parents facing similar situations in handling childhood ED.

The results presented in this article are part of a larger study exploring mothers’ perceptions of raising a child with ED, how childhood ED personally impacted family members, and how parents managed day-to-day challenges related to their child’s ED. It is beyond the scope of this article to present the entire results of this wider study. Data sharing mothers’ experiences during the diagnosis process and mothers’ perceptions of the emotional family impact due to childhood ED are presented elsewhere [4]. In this prior publication, mothers revealed difficulties they experienced trying to find effective parenting approaches to raising a child with ED [4]. The current article presents the strategies these mothers personally employed to manage their child’s emotional and behavioral outbursts as well as how mothers addressed the needs of their other children, their spousal relationship, and their own self-care. These strategies provide suggestions for novel parenting approaches for a child with ED as well as caring for the needs of the entire family. Nurses and healthcare providers can use the present findings to spark conversations and collaboration with parents and families of children with ED.

### 2.2. Recruitment

Institutional Review Board (IRB) approval was obtained prior to any recruitment or study processes (University of Texas Medical Branch IRB, #23-0049). Participants were recruited through descriptive flyers shared on national social media. Participation was limited to parents (biological, adoptive or stepparents) of children who had been diagnosed with emotional disturbance or who had significant behavioral issues for at least six months. To ensure participants’ experience was recent, their children must be younger than 24 years old and lived with the participant throughout their childhood. Although fathers were not excluded from the study, data saturation occurred after eight interviews with participants who were all mothers. Two mothers had two different children diagnosed with ED, so the final sample was eight mothers with 10 children diagnosed with emotional disturbance.

### 2.3. Informed Consent

Protection of participants’ rights and potential ethical concerns were extremely important. Interested potential participants contacted the researcher, who described the study and participant criteria. If participants still wished to proceed, details about the study, including a verbal consent script, were sent to the participants. The consent script was read aloud before the interview began, and emphasized that participants could decline any question for any reason or could end the interview entirely at any time. As part of the consent script, the author reminded participants that as a registered nurse, she is a mandatory reporter of child abuse in her state. Participants were informed that if any information caused concern that a child was being abused or neglected, the interview would be halted, and the researcher must report their concerns to the authorities. All participants continued with the interview after giving verbal consent, and there were no indications of any child mistreatment in any participant’s interview. All data was masked for confidentiality, and the original, unredacted data was securely locked and stored away from the research site.

### 2.4. Data Collection and Analysis

Data was gathered through semi-structured interviews, field notes, and reflexive journaling. A semi-structured interview guide was created to initiate topics and prompt participants to remember situations they may have encountered. The interview was not strictly guided, but allowed each participant to focus on the experiences and areas of family life that were most important from the participant’s personal view. Interviews were conducted via recorded sessions over Zoom (San Jose, CA, US) or by telephone. Basic demographic data, including the participants’ age and their child’s age, was collected first. The interviews began with the phrase: “I am interested in learning about the lives of parents of children with emotional or behavioral difficulties. When did you start noticing things that caused you to think your child may have an emotional or behavioral problem?”. Participants moved through their story at their own pace. Additional data was collected by field notes, which documented the participant’s non-verbal reactions or impressions after each interview, and a reflexive journal was used to monitor any potential researcher bias.

Interview recordings were transcribed by an artificial intelligence transcription service (Otter.ai, https://otter.ai/). The researcher verified each transcript for accuracy by listening to the recording and correcting any transcription errors. All potentially identifying information about participants and their family members was masked and de-identified. Only masked data was used for data analysis. The original, unmasked transcripts, recordings, and participant contact data were securely locked and stored in a separate location.

Data analysis was conducted using the constant comparative method, originally proposed by Glaser and Strauss and further described by Lincoln and Guba [36]. Data analysis is done after each interview, comparing the results with prior interviews to refine interview topics and evaluate for data saturation. Phrases such as “what am I really looking for?” and “Now, I try not to take it personally” were moved into a category called “consciously changing reaction habits”. This category was eventually bridged with other actions mothers used to transform themselves and their own perspectives.

Microsoft Excel (2024/Microsoft 365) was used to store, sort, and re-sort data units into columns of groupings, and eventually into themes. Data were gathered until there was data saturation or “informational redundancy”. In NI, informational “redundancy” is defined as when subsequent interviews reveal scant or insignificant new information [36] (p. 234). Redundancy was suspected after seven interviews and was confirmed after eight interviews; therefore, further recruitment and interviews were halted.

### 2.5. Rigor

Rigor and trustworthiness in qualitative research are highly important, as rigor is required to determine if study results are “worth paying attention to” [36] (p. 290). There are four pillars of rigor in qualitative research to provide evidence of trustworthiness: credibility, transferability, dependability, and confirmability [36]. Several study processes were used to demonstrate rigor. Credibility was demonstrated through data triangulation between participant experiences. Transferability of the findings is enhanced through a rich description of participants’ stories and illustrative quotes. Dependability and confirmability were strengthened through peer debriefing, an audit log, and creation of a findings codebook, which tracked specific participants with common experiences.

The researcher’s positionality as a registered nurse was examined through peer debriefing and keeping a reflexive journal. In general, the public regards nurses as highly trustworthy professionals [38], which could increase participants’ comfort during the interviews. However, the researcher needed to ensure the interviews remained as data collection sessions rather than evolving into a caregiving or advising role. The nursing role perspective was also carefully considered during data analysis to mitigate potential bias as well as strengthen study credibility and confirmability.

## 3. Results

### 3.1. Sample Profile

The final sample comprised eight mothers (Table 1). Mothers’ average age was 48 years (29–62). Nearly all the mothers (seven of eight) had multiple children, with family sizes ranging between one and four children. The sample was mostly Caucasian (seven of eight), and one mother was Hispanic. Both biological and adoptive mothers were included. Five mothers had only biological children, one mother had only adopted children, and two mothers had both biological and adoptive children. The participants were from the United States but geographically widespread, with participants from both the east and west coast, and participants within the mid-west region of the United States.

The mothers raised 10 children with emotional disturbance, as two mothers had two different children with ED in their families. The children ranged from five to 23 years old. Six were biological children, and four were adoptive children. Most of the children (8 of 10) lived with other siblings at home. All the children were officially diagnosed with ED by a health or education professional. The children’s emotional disturbance caused various types of outbursts or challenges, including being highly overactive, emotionally volatile, unpredictable, and extremely inflexible. Many children displayed huge emotional reactions to any changes in schedules, routines, or environment, even when the change was minor or outside of the mother’s control. A few children could be violent to siblings or peers, or engaged in criminal acts. The children’s behaviors are summarized in Table 2.

### 3.2. Thematic Findings

The study used Lincoln and Guba’s Naturalistic Inquiry as a guiding qualitative methodology to better understand how mothers raising a child with emotional disturbance (ED) managed the everyday challenges caused by their child’s condition. As mothers’ stories and experiences were compared and analyzed, several commonalities were found amongst the participants. “**Responding to the challenges**” outlines the specific actions that mothers use to manage their child’s emotional disturbance at home. Mothers found that direct actions were usually less effective than adapting the child’s environment and their own reactions. “**Adaptations over time**” describes how mothers’ approaches, perspectives, and responses changed as they came to terms with their child’s condition and their child matured. Mothers realized the world looks very different through their child’s eyes, and seeing their child’s perspective had a dramatic effect on mothers’ personal perspectives. Mothers also engaged in specific “**self-care actions**”, which included recognizing the need for connecting to other people as well as consciously changing their own emotional reactions. The thematic findings are summarized in Table 3.

### 3.3. Responding to the Challenges

Mothers attempted an exhaustive number of actions and adaptations trying to moderate their child’s emotional and behavioral reactions. Mothers spent significant time and energy searching for parenting information and options to try. Mothers learned that direct actions or external consequences were unlikely to be effective, but that adapting both the environment and the mothers’ internal reactions achieved more positive results.

**Consistent consequences**. Initially, all the mothers tried stricter rules with consistent consequences. Many mothers called this “old school” parenting, which they experienced themselves growing up. It was consequences-based: “this is the rule…if you don’t follow the role, this is your consequence”. However, an authoritarian approach or consequences such as time-outs, grounding, yelling/anger, or spanking were completely ineffective at improving their child’s behavior.

*“Yelling at her, or if I was to hit her, does not produce the same response that it produced in me as a child. It just keeps her anger heightened.”*

Attempts to persuade the child to “make better choices” backfired, generally ramping up the child’s emotions instead of calming them down. None of the mothers found that consequences-based and traditional parenting was successful for any of their children with ED, even when the style succeeded for their children without ED.

**Expert assistance.** All eight mothers sought expert assistance, which included therapy (8/8), teachers /educational professionals (8/8), medications (4/8), and residential placements (4/8). Interactions with professionals, especially schools, had mixed results. Five children required temporary residential placements, with some requiring multiple stays. Although one mother felt the stay helped her child, other mothers said the residential stays were “a terrible experience” or that after their child came home, she would “go right back, within months, to the same behaviors”. However, residential placements did protect family members from violence and emotional chaos, giving families “an opportunity to breathe a little”, but rarely resulted in lasting improvement for the children themselves. Mothers tried individual and family psychotherapy, occupational therapy, play therapy, group therapy, and speech therapy. Finding a therapist who was both available and a good match for their child was time-consuming, impacting mothers’ jobs and their ability to care for their other children. One child’s speech therapy treatment was delayed for more than six months. In response, this mother taught herself speech exercises from the internet so she could give speech therapy to her child herself.

Every mother sought help from their child’s school, but their experiences were widely divergent. Schools were either their strongest ally or their worst battleground. Most of the parents (7/8) secured disability accommodations and protections for their child within the school system. Four mothers (with five children with ED) praised schools and teachers as “saints”. Mothers felt these teachers tried to understand their child, found creative ways to work around their child’s challenges, and saw their child’s positive aspects. Stellar teachers might create their own accommodations to relieve a child’s anxiety, or “loved the challenge” of successfully working with the child with ED. When mothers had strong relationships with their child’s teacher, they collaborated to find new ways to positively interact with their child. In positive school environments, mothers leveraged the power of their child’s Individual Education Plan (IEP) to become truly individualized and much more effective.

*“That’s when the accommodations would really get good. The teacher gives 50 problems to do. Does she really need 50 problems? The counselor asks, ‘What [number] do you think you can do?’ Then the school would change the lesson plan, which was a great relief to me.”*

For the remaining four mothers, schools were their worst battleground. Frequent staff turnover wrecked IEPs or delayed services. One administrator directly refused to follow the child’s IEP accommodations. This mother struggled to find any compromises with her child’s school, who wanted her child to do the same curriculum, in the same manner, and who wanted her child to come before school, during recess, during lunch, after school, and still had homework until 8:00 at night.

*“Could you give him less questions? Could you make sure he gets the crux of what the item is, you know? Can we can we not, you know, have him have to write it all out, because it’s never going to come out correctly… Can he, can you use some of the assistance (assistive devices) things? And it would just be, ‘He’s fine. It’s fine. He can just come on after school.’ He spent most of his time coming in after school to … make up for whatever it was. It was, ‘This is the curriculum, there’s nothing we can really do.’ “*

His mother sold their home, which uprooted all of her children, so the child with ED could attend a school that followed his IEP accommodations. Other mothers were horrified to discover teachers physically abused their child by hitting or kicking them, called their dyslexic child “stupid”, or forced the child to run for an hour while other children watched. Another teacher informed one mother that her daughter was being kicked by the teacher in class. When the mother asked her child about the situation, her daughter confirmed it happened, but she thought she deserved the kicking because she was a “bad girl”. In each abusive situation, the child did not inform their parents but simply suffered abuse in silence.

**Adapting the child’s world.** Mothers started adapting their child’s school, home environment, diet, and living arrangements to best suit the child’s needs, instead of constantly trying to change the child’s behaviors to fit in with society. Several mothers altered their personal expectations, so instead of constantly trying to get their child to fit into the world, they focused on adapting their world to better fit their child.

*“I know that when I take her to church, we’re going to be up and down and up and down and go to the bathroom, and coming back in and go the baby room and get up and coming back in… So, we try to take a seat that’s towards the back and away from other kids.”*

Mothers learned how heat or diet affected their child. One child had an inflammatory response to gluten foods or to being overheated. Once the mother discovered that pattern, she adapted her child’s exercise pattern, family diet, and even found that the occasional ibuprofen could dampen the inflammation and reset her child’s emotions fairly quickly, if taken early in an emotional episode. Physical activities could be very useful, and mothers became swim parents, equestrians, hikers, and campers.

*“I was able to be involved in activities…If it’s important to you, it’s important to me…girl scouts, band, swimming.”*

Some adaptations were life-altering and affected the entire family. Mothers changed jobs to ensure they increased their availability to respond to emergencies. Three mothers moved their families so their child could attend a different school or be nearer to supportive services. Other times, the child’s siblings were sent to live with relatives until the emotions were more stabilized. Another mother found online schooling to be the best fit for her daughter. This was a revelation for her because online schooling’s inherent flexibility allowed her to adjust to her child’s current emotional state. This mother emphasized, “it’s always been finding the environment for [my child] to thrive in”.

**Mothers adapting themselves.** As mothers learned that adaptation was a more successful approach, they adapted themselves. Most mothers (7/8) switched from an authoritarian- or consequence-based parenting style to an intentional, pre-planned parental response. Instead of reacting to the child’s current behavior or emotion, mothers emphasized natural negative consequences of the child’s actions, without adding extra punishment. One mother called it “responding with intentionality.”

*“We don’t respond to the outburst. We respond with intentionality to make sure that he understands that there’s safety and that there’s a schedule or that there’s boundaries.”*

For example, when a child threatened to destroy her art in a fit of rage, her mother said very evenly, “[Your art] will get yucky in the garbage, because there’s old food in there”, rather than getting angry or trying to convince the child not to destroy it. Mothers learned their child was incapable of listening in the heat of the moment: “You cannot reason with her until she is finally calm”. Therefore, defusing emotions became the primary goal, while responding to the situation became secondary. Mothers leaned on natural consequences over imposed punishments. If a child got a poor grade, was suspended, or even arrested, the consequence stood for itself. Parents did not avoid or add to the natural consequence, which allowed parents to emotionally detach themselves from the emotional chaos.

Mothers changed their communication styles, praising small but good decisions within an overall outburst, deliberately avoiding battles, and defusing emotions. Intentionally avoiding arguments tended to resolve the conflict more quickly, similar to playing tug-of-war, but then deciding to drop the rope: “If I stop arguing, usually I have better results”. Mothers of older children were more successful when they started the conversation by empathizing with the child’s view, rather than giving their own perspective. One mother recalled how her conversations with her daughter improved when she changed her approach.

*“I said to my daughter, ‘I know you feel that way, but I promise you it’s not. I know, it’s okay to feel and it’s okay to feel that way. But everybody has their struggles. Just remember that we’re all struggling with something.’ So, I’m not saying things [to her] like ‘get over it,’ you know, or ‘at least you’re still on the team.’ You know, I didn’t say things like that. Like I probably would have said [to her] when she was a little bit younger.”*

Mothers’ intentional, pre-planned responses did not prevent their child from having outbursts. However, intentional responses helped mitigate turmoil, defuse emotions more quickly, and improve overall communication with their child.

### 3.4. Adapting over Time

**Seeing the world through their child’s eyes.** As mothers switched to these intentional, pre-planned responses, they started seeing the world differently. All eight mothers discussed moments when they saw how the world appeared from their child’s perspective. They realized their child did not see invisible social rules, had very rigid beliefs about “fairness”, or missed the nuances within rules. Mothers realized how many times the social rules they tried to teach their child had implicit caveats. For example, when a mother learned her child was being kicked by her teacher, she also learned why her daughter did not report the abuse. From her daughter’s point of view, she had misbehaved; therefore, punishment was merited, and so she simply suffered the abuse in silence. The child could not see the nuances between being respectful to a teacher and accepting consequences, and that this teacher’s “punishments” were wrong and should not be accepted. A child with ED may struggle to navigate the differences between the rules of “respecting” authority figures and necessary self-advocacy, whether they are asking for extra time on a test or reporting physical abuse. Another child did not know it was abnormal to be anxious every minute of the day.

*“She [her daughter] didn’t know it wasn’t ok to feel like that….we found out that in her mind, she had been struggling her whole life. And so, she just knew how to mirror it, to look happy and do what she needed to do.”*

Mothers realized their child often did not know implicit social rules, but knew if they were failing their parents. They realized their child was trying to conform.

*“They’re holding it together as best they can at school, and they come home, and they just let loose [gestures like an explosion]…they know this is how we’re supposed to behave, so we’re trying really hard to conform.”*

Seeing how the world looked through their child’s eyes was shocking, sobering, and sad for mothers.

**Mothers’ perspective changes.** Over time, using intentional responses and seeing the world differently allowed mothers to see their child as someone *struggling with a problem*, and not just a child *being a problem*. They realized their child had a rationale for their over-the-top reactions. By seeing their child had a reason, even when mothers disagreed with the reason, mothers felt more understanding and compassion for their child. After one child got into a fight at school, her mother realized she had been bullied and injured the previous week by the same child, but was rebuffed by the teacher. The mother did not fight the natural consequence of her child’s school suspension, but told the principal: “I’m not saying what she did was right. But I’m just saying… that’s why she did it”.

Seeing their child’s perspective created compassion in other ways. Mothers realized their child was not being consciously or willfully difficult, but had a limited ability to control their own reactions. Mothers also realized their child had been silently suffering in front of them. One child with anxiety and depression realized that other children did not have those overwhelming feelings.

*“When she got older and filled out a thing about depression and how she felt. She goes, “I just thought it was normal to feel the way I felt. I didn’t know I was lying.” I just wish I had known so that when we had these battles, we would have been a little softer with her.”*

Mothers expressed regret that it took them so long to see how the world looked from their child’s perspective.

*“I wish I would have known earlier to maybe, you know, think about it from her perspective and start with compassion. Rather than start with authoritarian.”*

As mothers’ viewpoints changed, priorities shifted to the “here and now” issues and let go of “normal” expectations. One mother described it as a series of baskets: “A” for top priority (safety), then “B” for immediate concerns, and “C” for optional things. Priorities shifted as baskets emptied or filled. Another couple divided responsibilities into clearly delineated spheres. This couple divided up who would assist with which school subjects, who attended which meetings or appointments, and who attended which sporting events or performances.

*“Being really careful about boundaries, selection of me or my husband about who’s responsible for what meeting…we going to do what we can, when we can, divide and conquer. And we are going to let whatever happens…go”*

The transition in perspective also created space to see their child’s positive aspects more clearly. One mother proudly explained:

*“It just wasn’t in my mind to defy [authority figures], which can be detrimental in your later life, when you just going to…blindly follow and maybe get pushed around…The one thing I can say about [daughter] is that she’s not going to get pushed around. And she’s her worst critic, but she’s also pretty ferocious.”*

### 3.5. Mothers’ Self-Care

Mothers were asked what they did to sustain themselves while navigating the chaotic challenges associated with their child’s ED. Mothers engaged in several activities to cope during these difficult times. Two major themes surrounded the importance of connecting with other people and consciously changing their internal emotional reactions.

**Connecting with others.** All the participants stressed the benefit of connecting to other people to combat their feelings of isolation. Married mothers treasured their spouses’ support as their “best friend. One mother described a verbal pact she made with her spouse.

*“We made agreements. Basic agreement is whatever decision you make, you’ve made the best decision you can make, we will never argue about a decision somebody else makes. And that conversation happened with my spouse.”*

Mothers also stressed the importance of sharing their challenges with friends, church members, and co-workers. One mother said the best connections came from friends who called to say, “How are you? Like, how are *you*? Not just, how are things”. Finding other parents dealing with similar problems was extremely helpful.

*“Over the years you find those other parents that are having those same struggles. So, you make your own little group of friends.”*

Connections might come with attending church, going to work, or volunteering. Mothers felt these connections were the best survival tips they could give to other parents who may be struggling with their own child with ED. Sometimes, friends or family members’ lack of support was shocking, disappointing, or hurtful. One negative case occurred when a mother revealed her daughter’s issues to other church members. She was surprised that church members had noticed- but did not mind- her daughter’s antics in church. She wondered if the stigma was stronger in her own mind than in others’. Overall, mothers encouraged others to keep being open, despite the risk, because they often gained tips and suggestions when they least expected them.

*“Parents, you just have to be open. And it’s not scary. It seems scary. But the more you know, the more you can help them [your child].”*

*“What works for you, may not work for them, but just have that openness to be like, you know what, I’m just gonna listen. Or…I don’t think that’ll work, but I could do this and tweak it a little and that might work.”*

**Changing mental reactions**. Lastly, mothers spend a lot of effort changing their initial emotional reactions to their child’s behaviors. One example that several mothers used was focusing on the “now” problems to avoid being too overwhelmed by future, hypothetical worries.

*“[researcher: how did you manage?] [participant sighs] That’s a good question. [starts laughing] I don’t know. It was, yeah, I don’t know. Day at a time, sometimes it was a minute of time.”*

By focusing on the “now”, it was easier to ride out the natural highs and lows that come with raising a child with ED. Mothers spoke about riding the waves of chaos, because “one day it might be one thing, and the next, it’s completely different”. Mothers released their preconceived “normal” mothering ideals to focus on mothering the child they have. Mothers who divided responsibilities said, “We’re going to do what we can, when we can, we’re going to divide and conquer. And we are going to let whatever happens…go”. By constantly re-focusing on the present moments, mothers could enjoy the good times, remember the love of their spouses and friends, temporarily break their cycle of stress, and renew their own sense of purpose.

## 4. Discussion

This study revealed several strategies that mothers raising a child with ED used at home to manage their child’s emotional or behavioral issues. Mothers spent enormous amounts of time and energy searching for potential solutions and parenting methods. Although Naturalistic Inquiry theorists advocate for creating a composite of participants’ realities rather than generating an explanatory theory or model [36] (p. 335), the study’s findings could guide future interventional research to address the self-care deficits described by these participants. Orem’s self-care deficit theory and Taylor’s theory of family and dependent care deficits provide a basis for researching the applicability of the strategies used by these mothers [39]. As data saturation was achieved before fathers had participated, certain limitations of the experiential findings must be acknowledged. However, given the lack of information pertinent to the challenges parents face at home, these ideas have practical implications for healthcare providers working with parents of children with ED.

Foremost, mothers here found that traditional, consequences-based parenting techniques consistently failed when applied to a child with emotional disturbance, even when consequences-based parenting worked with their other children. Changing parenting methods is not easy, and one mother admitted she and her spouse “were struggling with the idea that we have to parent her differently than how we were parented”. It is difficult to know if reactionary, consequences-based parenting may be a cultural expectation within this small, ethnically similar sample. Traditional parenting advice from child development experts is often based on Erikson’s stages of development [40], but these mothers found consequence-based parenting responses to be ineffective. If healthcare providers rely on traditional parenting advice, such as that from Erikson’s theories, they may not be able to truly assist parents of children with ED.

This study found that adapting the child’s environment, prioritizing emotional diffusion over behavioral reactions, emphasizing natural consequences, and adapting mothers’ emotional reactions were more likely to be successful. Mothers found using intentional responses, rather than reactions, were not only more effective but also allowed mothers to emotionally detach from arguments, reducing their own stress. A literature review conceptualizing emotions within the parent–child bond indicated that creating supportive environments and tailoring parental responses to a child’s emotional sensitivity could accelerate a child’s emotional self-regulation [41]. Pediatric healthcare providers might consider using these findings as potential suggestions for parents seeking advice for the child’s behavioral issues. Primary pediatric healthcare providers could use the findings to spark conversations with parents struggling with a child with ED. Even if the specific idea is unfeasible, the conversation itself may spark greater creativity, collaboration, and a feeling of partnership between parents and healthcare providers. Other families, as well as healthcare and educators, may benefit from these unique approaches to problematic behaviors.

Mothers experienced widely divergent responses from schools and educators. On one hand, four of the eight mothers received their strongest expert support from their child’s teachers. These parents found collaborating with schools to be extremely rewarding. Mothers fostered wonderful collegial relationships between the parent and the teacher, which ultimately benefited the child immensely. Mothers who were allowed to create truly individual education plans praised the collaborative flexibility, which still ensured their child’s education. The relationships demonstrated between these mothers and schools should be further shared as examples for other parents and schools to strive towards. However, the injustices and abusive practices experienced by three mothers are extremely troubling. Schools currently face great internal and external pressures to handle larger classrooms with fewer resources but greater scrutiny. School activities such as recess, the fine arts, and physical education are replaced with increasing rigor in the classroom. To keep up with a more rigorous curriculum, teachers may expect parents to offer greater assistance with homework and follow up at home [42]. In addition, schools may lack teachers with specialized training to handle children with behavioral issues or face funding inequities for proper school staffing. Teachers may assume that parents have more “control” over their children at home than parents actually do.

The limitations of a small sample make interpretation and transferability of the school experiences difficult. Participants in this study were geographically widespread, so these findings were not regional in nature. The study was designed to explore the home setting, not schools, but participants’ experiences were so divergent and concerning that this area should be explored further in future studies. One possible approach could be utilizing school nurses to bridge communication between teachers and parents. School nurses’ educational background regarding pediatric development and mental health issues could assist both parents and teachers, but not all schools employ school nurses. Regardless, the findings deserve greater attention to bridge the gaps between parents and teachers for improved partnership and collaboration.

An important turning point occurred when mothers saw the world from their child’s perspective. Some children suffered mistreatment in silence, and their reactions were unpredictable, extreme, or illogical. Discovering the presence of rationales behind their child’s outbursts was significant, even if the child’s rationale was warped or if the outburst was extreme. When mothers learned their child had rationales that prompted their (over)reactions, mothers viewed their child’s explosion as an outward sign of their child’s internal struggle. This shift allowed mothers to see their child’s struggle as worthy of compassion, instead of only causing the mothers frustration.

Parents seek advice from pediatric healthcare providers [2,3,16,17], and this perspective shift is an opportunity for nurses and healthcare providers to support parents more effectively. Other parents may learn that their overreacting child may also be suffering. These findings support the benefit of mindfulness and self-compassion found by others [15,28]. Family self-care deficits could be reduced by assisting parents to see their child’s outbursts from their child’s world-view perspective [39]. Healthcare providers could facilitate perspective shifts by actively looking for potential rationales that a child might not verbalize. Since healthcare providers are rarely engaged in direct conflicts with the child themselves, it may be easier for providers to consider an alternative point of view and encourage parents in this journey.

Lastly, these mothers addressed the importance of connecting with others to combat their own feelings of loneliness, isolation, and stigma. These prior findings indicate that stress was exacerbated by isolation [2,4,13]. These mothers revealed that despite their fear of rejection, finding others to talk to was worth the risk. European studies examining parenting interventions [22,23,24] have a destigmatizing effect by their very existence, and replication of similar parenting interventions could assist parents in the U.S. both in terms of learning parenting approaches and mitigating isolation. Prior pilot programs appeared to be well received [27,29], and mothers here struggled to find similar programs. Pediatric healthcare workers should encourage mothers to keep searching for connections, whether the connections be family, friends, work, or even online support groups.

### Limitations

As an exploratory study, the findings must be interpreted with caution due to the limitations of a small and homogenous sample. Data collection was conducted over online meetings, which allowed for greater geographic sampling. The sample was geographically diverse, with participants from both coasts of the United States as well as the far north and south mid-region. However, the sample was all women and ethnically similar, with only one Hispanic participant and the rest were Caucasian. The study criteria did not limit participation to mothers, but data saturation was achieved before any fathers participated. This is an important limitation, as fathers’ perceptions are likely to be very different. In addition, the age distribution amongst the 10 children was uneven. Three children were under age 10, and the remaining seven children were 15 or older. One mother of a 20-year-old remarked she could not have participated when her child was in her early teens, because her behaviors were much worse then. We need to learn what strategies could be applied to children in early adolescence.

Despite these limitations, the study still provides new data regarding parents’ experiences at home raising a child with ED. Future studies should be conducted to gain more gender and ethnic diversity, as well as a broader sampling, to confirm these findings. Schools’ responses to children with ED, policy guidance for educators, and the success of strong programs are areas that deserve more focused future research. Lastly, interventional studies could test the effectiveness of deliberate adaptation of parenting responses and perspective to both lessen parental stress and improve the child’s emotional regulation.

## 5. Conclusions

In this study, mothers shared vivid details about their parenting experiences raising a child with emotional disturbance (ED). The findings demonstrate the need to consider the challenges of childhood ED as a family-centered phenomenon, rather than as a child-centered problem. Mothers tried an exhaustive list of internal and external adaptations to manage common challenges that arose from their child’s emotional disturbance. Although any single action or adaptation used might be beneficial for any child, these mothers of children with ED found that adaptation became absolutely essential to managing the volatility of their child’s behaviors and reactions. As their child matured, mothers were able to see their child’s view of the world and found their perspectives changed. In addition, those mothers who adapted their own emotional reactions found that they became more peaceful and compassionate toward their child in the process. This study should be used to guide further investigations of childhood ED for potential interventions and parenting advice. This data will help to improve not only the health and outcome of the child with ED, but also the health and well-being of the whole family.

## Figures and Tables

**Table 1:** Sociodemographic data: Mother participants (n = 8).

Age	Mean = 48 Years	Range = 29–62	
Marital Status	Married = 6	Divorced = 1	Single = 1
Ethnicity	White = 7	Hispanic = 1	
With adopted or biological children?	Adopted children only = 1	Biological only = 5	Mother with both biological and adopted children = 2

**Table 2:** Sample: Behavioral problems of mothers’ children with emotional disturbance (n = 10).

Age Group (Years) Gender	Aggregate Behaviors
5 to 9 years 2 Females, 1 Male	Extreme reactions to environmental changes Extreme reactions to peers’ actions Inconsolable crying spells, lasting hours Rigid rules about meals or morning routines Flies into a rage, attacking peers or adults Runs away from school or in stores Hyperactive
15 to 19 years 3 Females, 1 Male	Extreme anxiety to be the “best” Intolerant of peers not following rules Seemingly unaware of social expectations Emotionally volatile, screaming outbursts at family Violent outbursts required police intervention Criminal behavior (theft, armed robbery)
20 to 23 years 2 Females, 1 Male	Extremely physically active Fights at school frequently Panic attacks, self-harming Screaming attacks at school Emotionally volatile Unable to read social cues

**Table 3:** Summary of themes: How mothers managed childhood ED.

**Responding to the Challenges**
*Consistent Consequences* Very much what I would say the old school way, like, this is the rule. You follow the rule. Don’t follow the rule, this is your consequence. It does not produce the same response it produced in me as a child. She is…defiant. And it just keeps her…heightened.
*Expert Assistance* That’s when the accommodations would really get good. The teacher gives 50 problems to do. Does she really need 50 problems? The counselor asks, “What [number] do you think you can do?” Then the school would change the lesson plan, which was a great relief to me.
*Adapting to the Child* There’s certain settings that we know that we can take him and if we’re gonna go out to the apple orchard where he can just run free. That’s a better experience for him than going out to dinner with everyone. It’s always been about finding the environment for my child to thrive in.
*Mothers Adapting Themselves* We don’t respond to the outburst. We respond with intentionality to make sure that he understands that there’s safety and that there’s a schedule or that there’s boundaries
**Adaptations over Time**
*Seeing Through their Child’s Eyes* She didn’t know it wasn’t ok to feel like that. We found out that in her mind, she had been struggling her whole life. And so, she just knew how to mirror it, to look happy and do what she needed to do.
*Mothers’ Perspective Changes* I wish I would have known earlier to maybe, you know, think about it from her perspective and start with compassion. Rather than start with authoritarian.
**Mothers’ Self-Care Actions**
*Connections with Others* Keep trying to find others that you can talk to…you need to be able to vent. Trying to find some of those people, so you are not alone
*Changing Mental Reactions* Being really, really careful about boundaries, selection of between me and my husband of who’s responsible for what meeting. Who’s doing [what] the breakdown of what’s happening. We’re going to do what we can, when we can, we’re going to divide and conquer. And we are going to let whatever happens…go

## Data Availability

Data which supports these findings are available on reasonable request. Due to privacy considerations, data has been anonymized and will only be shared with appropriate safeguards.

## Abbreviations

The following abbreviations are used in this manuscript:

ED	Emotional Disturbance
DoEd	Department of Education
US	United States

## Appendix A. Narrative for Oral Consent

As we’ve discussed before, the purpose of this study is to gather information about the experiences and challenges that parents of children with emotional disturbance encounter, coping strategies parents have tried, and how parents managed those challenges.

I am asking parents of children with emotional or behavioral problems to share their experiences about how raising a child with these problems has affected you and your family. I am interested in researching this issue so healthcare providers and educators can better understand what parents raising these children deal with and need for support. I am conducting this study as part of my doctoral dissertation research at the University of Texas Medical Branch.

You have indicated you are a parent who is currently raising (or have raised) a child (or children) with long-term emotional or behavioral problems, and these problems have been present or lasted longer than six months.

Your participation in this study will consist of at least one interview, which will not last longer than 90 min, and potentially one or two follow-up interviews, which will not last longer than 30 min. This study is expected to pose very little risk to anyone who participates.

Potential risks of the study include a possible loss of privacy or confidentiality, and possible feelings of fatigue or distress.

Your privacy and confidentiality are very important. To protect your confidentiality, I will substitute a number for your name, and any information stored about you will only have that number as identification. I also will remove any names or other identifying information, such as the name of a school, from your information. No one will see or hear this recording except myself or my research team.

Some parents may find talking about or remembering challenging times with their child to be stressful or tiring. If at any time during this interview you feel fatigued or emotionally upset, please let me know. We can pause the interview for a break, reschedule for another day, or stop the interview completely, whichever you prefer.

I plan to ask you demographic questions about you and your immediate family, such as age, gender and grade level in school. Then I will ask you about the challenges you and your family have dealt with surrounding your child with the emotional or behavioral problems.

For some participants, the opportunity to share their parenting experiences raising children with emotional or behavioral disorders can be very positive event and allow parents to feel “heard”. There may be a benefit to other parents and society in general to learning how parents of children with emotional or behavioral disorders, like yourself, cope and manage the day-to-day challenges these children present. However, there is no other direct benefit to you personally from your participation in this research project.

Your participation is entirely voluntary. You can choose not to answer any question at all, or stop the interview completely at any point, for any reason you wish, and I will respect your wishes. You can withdraw from this study at any time during the interview or even after the interview, and I will respect your decision and remove your information completely.

The following information is very important. As a nurse, I am required by law to report any suspected child abuse or neglect (or abuse/neglect of an adult who is disabled). In Texas, these reports must be made to the Texas Department of Family and Protective Services. I will not be asking you any questions about abuse, but if any information is revealed during the interview that creates a concern for potential or ongoing abuse or neglect of a child or disabled adult, I am required by law to notify the authorities. At that point, your confidentiality will need to be broken so that I can report the issue to the proper authorities. If that were to happen, I would let you know immediately.

Do you have any questions about this study or participating in this study? (Researcher will now pause and answer any questions the participant raises. Once all the participant’s questions are answered to the participant’s satisfaction, the researcher will proceed with the following question.)

At this point, I would like to directly ask you if you agree to participate in this study. If you say “no”, I will not start the recording, and your information will be not used in this research study. If you say “yes”, I will begin the recording. I will ask you to repeat your agreement on the recording, and then start the interview by asking you the demographic questions.

Do you agree to participate in this study?
